# Optimizing the Production of Nursery-Based Biological Soil Crusts for Restoration of Arid Land Soils

**DOI:** 10.1128/AEM.00735-19

**Published:** 2019-07-18

**Authors:** Julie Bethany, Ana Giraldo-Silva, Corey Nelson, Nichole N. Barger, Ferran Garcia-Pichel

**Affiliations:** a Center for Fundamental and Applied Microbiomics, Biodesign Institute, Arizona State University, Tempe, Arizona, USA; b School of Life Sciences, Arizona State University, Tempe, Arizona, USA; c Department of Ecology and Evolutionary Biology, University of Colorado, Boulder, Colorado, USA; Wageningen University

**Keywords:** 16S rRNA, biocrust, biocrust restoration, biological soil crust, cyanobacteria, degraded soils, drylands, erosion control, restoration, soil microbiome

## Abstract

Biological soil crusts (biocrusts) are topsoil communities formed by cyanobacteria or other microbial primary producers and are typical of arid and semiarid environments. Biocrusts promote a range of ecosystem services, such as erosion resistance and soil fertility, but their degradation by often anthropogenic disturbance brings about the loss of these services. This has prompted interest in developing restoration techniques. One approach is to source biocrust remnants from the area of interest for scale-up cultivation in a microbial “nursery” that produces large quantities of high-quality inoculum for field deployment. However, growth dynamics and the ability to reuse the produced inoculum for continued production have not been assessed. To optimize production, we followed nursery growth dynamics of biocrusts from cold (Great Basin) and hot (Chihuahuan) deserts. Peak phototrophic biomass was attained between 3 and 7 weeks in cold desert biocrusts and at 12 weeks in those from hot deserts. We also reused the resultant biocrust inoculum to seed successive incubations, tracking both phototroph biomass and cyanobacterial community structure using 16S rRNA gene amplicon sequencing. Hot desert biocrusts showed little to no viability upon reinoculation, while cold desert biocrusts continued to grow, but at the expense of progressive shifts in species composition. This leads us to discourage the reuse of nursery-grown inoculum. Surprisingly, growth was highly variable among replicates, and overall yields were low, a fact that we attribute to the demonstrable presence of virulent and stochastically distributed but hitherto unknown cyanobacterial pathogens. We provide recommendations to avoid pathogen incidence in the process.

**IMPORTANCE** Biocrust communities provide important ecosystem services for arid land soils, such as soil surface stabilization promoting erosion resistance and contributing to overall soil fertility. Anthropogenic degradation to biocrust communities (through livestock grazing, agriculture, urban sprawl, and trampling) is common and significant, resulting in a loss of those ecosystem services. Losses impact both the health of the native ecosystem and the public health of local populations due to enhanced dust emissions. Because of this, approaches for biocrust restoration are being developed worldwide. Here, we present optimization of a nursery-based approach to scaling up the production of biocrust inoculum for field restoration with respect to temporal dynamics and reuse of biological materials. Unexpectedly, we also report on complex population dynamics, significant spatial variability, and lower than expected yields that we ascribe to the demonstrable presence of cyanobacterial pathogens, the spread of which may be enhanced by some of the nursery production standard practices.

## Abstract (precis)

Biocrust communities provide important ecosystem services for arid land soils, such as soil surface stabilization promoting erosion resistance and contributing to overall soil fertility. Anthropogenic degradation to biocrust communities (through livestock grazing, agriculture, urban sprawl, and trampling) is common and significant, resulting in a loss of those ecosystem services. Losses impact both the health of the native ecosystem and the public health of local populations due to enhanced dust emissions. Because of this, approaches for biocrust restoration are being developed worldwide. Here, we present optimization of a nursery-based approach to scaling up the production of biocrust inoculum for field restoration with respect to temporal dynamics and reuse of biological materials. Unexpectedly, we also report on complex population dynamics, significant spatial variability, and lower than expected yields that we ascribe to the demonstrable presence of cyanobacterial pathogens, the spread of which may be enhanced by some of the nursery production standard practices.

## INTRODUCTION

Biological soil crusts (“biocrusts”) are topsoil microbial communities that include populations of cyanobacteria or microalgae as primary producers (1), as well as bacteria (2), archaea (3), and fungi (4) as heterotrophs. In some well-developed biocrusts, mosses (5) or lichens (6) are also primary producers. Biocrusts generally occur in the interspaces between plants throughout arid and semiarid environments and provide a variety of ecosystem services. Among them is the mitigation of erosion due to the action of pioneer filamentous nonheterocystous cyanobacteria, such as those in the genus *Microcoleus*, that adhere to and stabilize soil particles due to their large size (7) and polysaccharide sheath (8). Biological soil crusts fix and release key nutrients, such as carbon (9, 10) and nitrogen (11), and a variety of other micronutrients (12) that increase soil fertility.

In spite of their remarkable resilience to climatic extremes, biocrusts are subject to damage and even destruction by compressional stress associated with anthropogenic activities such as agriculture, especially livestock grazing (13), and recreational activities (14). Large portions of arid lands are impacted by these pressures and have become devoid of their once natural biocrust cover. Damaged lichen and moss biocrusts revert to the simpler assemblages characteristic of early successional stages with phototroph niche replacement by cyanobacteria (15). Natural recovery rates vary widely for biocrust communities, although generally, unassisted recovery is slow, with the most arid locations presenting the lowest rates (16). Unassisted full recovery can take from multiple decades to centuries (17, 18). Yet, compositionally simple cyanobacterial crusts can recover relatively quickly, in periods of months to several years (14, 19), if conditions are conducive to growth and propagules are present. This scenario has spearheaded a recent surge in attempts to actively restore biocrusts.

Early biocrust restoration attempts relied on transplanting intact biocrusts to crustless locations. While this proved that restoration was possible (20–22), it represents an unsustainable net-zero approach. Current alternative foci include inoculation with mass-cultured biocrust organisms, typically cyanobacteria (23–30) or mosses (31), and the so-called biocrust “mixed-community” approach (32), where a small amount of remnant biocrust from a disturbed site is used as a seed to grow large amounts of compositionally mixed inoculum in greenhouses or “microbial nurseries.” The advantages and shortcomings of each were recently discussed (33). The mixed community approach results in an inoculum that is (i) location specific, (ii) preconditioned to native edaphic factors, and (iii) amenable to quality control of microbial community composition. Optimally, conditions are set so that while overall growth is promoted, species composition is kept as close as possible to that of the field sites of origin. However, the effort associated with the mixed-community nursery approach is still subject to optimization. For example, aspects of temporal growth dynamics or the feasibility of utilizing the nursery-reared product as a sustainable seed for recurrent continuous production were not addressed in the original work (32). With this in mind, and according to the protocol established in the original work (32), we set out to first evaluate in detail nursery biocrust growth dynamics to minimize the incubation time needed to attain the biomass carrying capacity of particular soils as well as evaluate its variability among different soils. In a second objective, we wanted to test the possibility of reusing nursery-grown biocrusts for several growth rounds while maintaining high growth potential and a stable community composition, so as to further reduce the need for often meager field biocrust remnants.

## RESULTS

### Population dynamics.

Each of our four treatments consisted of two phases. Phase A was a time course designed to assess biocrust growth dynamics to minimize incubation times. We aimed to establish when nursery-grown biocrusts first reach biomass levels similar to those found in the field. Phase B was designed to determine if greenhouse-grown biocrusts could be reused to seed recurrent inoculum production. In both phases, nursery incubation conditions roughly mimicked the natural environment of origin in terms of temperature but were run with a watering regime and nutrient additions designed to maximize growth but minimize shifts in community structure, as determined previously (32).

**FIG 1:**
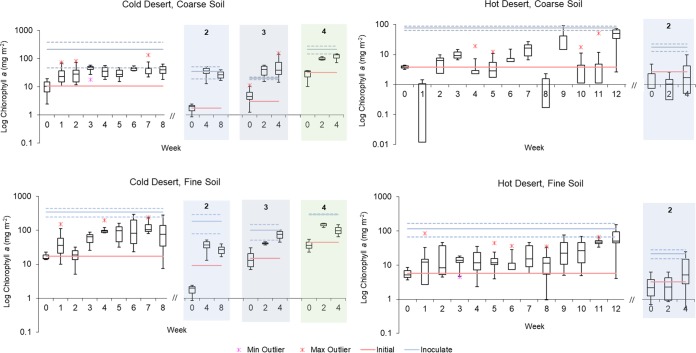
Growth dynamics of phototrophic biomass (as chlorophyll *a* areal concentration) during biocrust incubations. Phase A is shown on the far left of each panel. Successive phase B reinoculations are shown under shading and numbered. For each time point, box plots indicate upper and lower quartiles, and median values are shown as solid lines within the boxes. Whiskers denote upper and lower range, and asterisks denote outliers. For phase A, *n* = 6, except at *t* = 0, where *n* = 3. For reinoculation 2, *n* = 6 except at *t* = 0, where *n* = 3. For reinoculation 3, *n* = 4, and for 4, *n* = 3. Blue lines denote chlorophyll *a* content of field biocrusts used as original inoculum (*n* = 3), and red lines indicate chlorophyll *a* content at *t* = 0 (*n* = 3). Solid colored lines indicate mean values and dashed lines indicate one upper and one lower standard deviation of field biocrusts (*n* = 3).

In phase A, we expected that biomass would steadily increase with time, eventually reaching a carrying capacity typical for the particular soil and setting, with biomass remaining invariant thereafter. We also expected that the carrying capacity would be roughly similar to the biomass of established biocrusts in the field. In this scenario, establishing the minimal time required to reach this carrying capacity would be the main contribution of these experiments to process optimization. However, we found little support for these expectations in our experiments (Fig. 1). Overall, biocrust growth in phase A was quite variable among independent replicates, with chlorophyll *a* in individual containers widely varied at any time point. While the general trends of increasing chlorophyll *a* with time held for all incubations, the variability made it difficult to establish clear linear (or exponential) dynamics. In all treatments, the time course of chlorophyll *a* seemed to denote complex dynamics instead.

Peak average chlorophyll *a* was reached between weeks 3 and 7 for cold desert biocrusts but only at week 12 for hot desert biocrusts, after some events of significant chlorophyll *a* loss (Fig. 1). Yet, single independent containers showed very fast growth, attaining 10-fold increases in as little as 1 week, as can be seen in the single outlier point at week 1 in the fine, cold desert crust incubation.

In the cold desert coarse soils, chlorophyll *a* peaked at week 3 with an average level of 44 mg m^−2^ (*n* = 6) (Fig. 1), which was still below the initial value of the remnant field biocrust from the site. Otherwise, the dynamics of growth in this biocrust, with an initial steady increase followed by statistical stasis, was the only case that followed expected patterns. In the cold desert fine soils, chlorophyll *a* peaked at week 7, with an average of 130 mg chlorophyll *a* m^−2^ (*n* = 6, also significantly below its potential in the field), but the dynamics were not clearly differentiated from those of a slow steady increase. An apparent net loss of chlorophyll *a* occurred at week 2, but this was not significant and probably the result of high intercontainer variability. In both cold desert biocrusts, chlorophyll *a* differences from initial to peak were statistically significant (either Welch’s *t* tests, *t* = −5.08, *P* = 0.0005 for coarse soil, or Kolmogorov-Smirnov tests, D = 1, *P* = 0.005 for fine soil). Although chlorophyll *a* peaks were identified in both coarse and fine soils, these were statistically lower than their respective field levels (Welch’s *t* tests, *t* = −2.77, *P* = 0.04 and Kolmogorov-Smirnov D = 1, *P* = 0.005, respectively), indicating that the yield was below what was observed in remnant biocrusts.

In the hot desert soils, average chlorophyll *a* took a full 12 weeks to reach levels nearing those of the field. Here again, some individual containers were still not near chlorophyll *a* inoculum levels at this time. In the coarse soil, for example, chlorophyll *a* levels consistent with field levels were reached in only four of the six replicate containers, and in fine soils, only two biocrust containers reached that level. In spite of this divergence among replicates, differences in average biomass from time of inoculation to peak were statistically significant by week 12, according to Welch’s *t* test (*t* = −3.87, *P* = 0.01 and *t* = −2.88, *P* = 0.03; for fine and coarse soil, respectively). In fine soils, biocrust chlorophyll *a* levels were indistinguishable statistically from those of the field biocrusts (*t* = 1.66, *P* = 0.128), but in coarse soil, chlorophyll *a* was significantly lower than in the original biocrusts (*t* = 2.54, *P* = 0.04). The population dynamics in these hot desert soils was quite complex, with frequent outlier chlorophyll *a* values and with several cases of significant declines in chlorophyll *a* well below the level of inoculation (weeks 1 and 8 in the coarse hot biocrusts, for example).

Tracking of phototrophic growth from airborne cyanobacteria in control containers allowed us to exclude the possibility of contamination, since no controls resulted in any significant phototrophic biomass during phase A.

### Reinoculation potential.

Biocrusts obtained during phase A were pooled and used as inoculum for phase B, in which the biomass resulting from a round of growth was used to seed the next round. Biocrust in hot desert soils did poorly when using recycled inoculum. After 4 weeks of incubation, median coarse and fine soil biocrust chlorophyll *a* levels were in fact below inoculum levels. For this reason, trials were ended early (Fig. 1). In contrast, phototrophic biomass in cold desert containers recurrently attained significant levels within 4 weeks of incubation, although not always to target levels. Cold desert biocrusts from coarse soils in the 2nd and 3rd growth rounds produced chlorophyll *a* (median) levels that met or exceeded that of the inoculum (Fig. 1). Fine soil biocrusts did not reach inoculum levels in the 2nd growth round; however, with increased inoculum density, median chlorophyll *a* levels were within one standard deviation of inoculum levels after 4 weeks in the 3rd growth round (Fig. 1).

Again, tracking of phototrophic growth in control containers allowed us to exclude the possibility of contamination in all but the final round of cold coarse incubations (Welch’s *t* test, T = 4.30, *P* = 0.49).

### Community composition.

**FIG 2:**
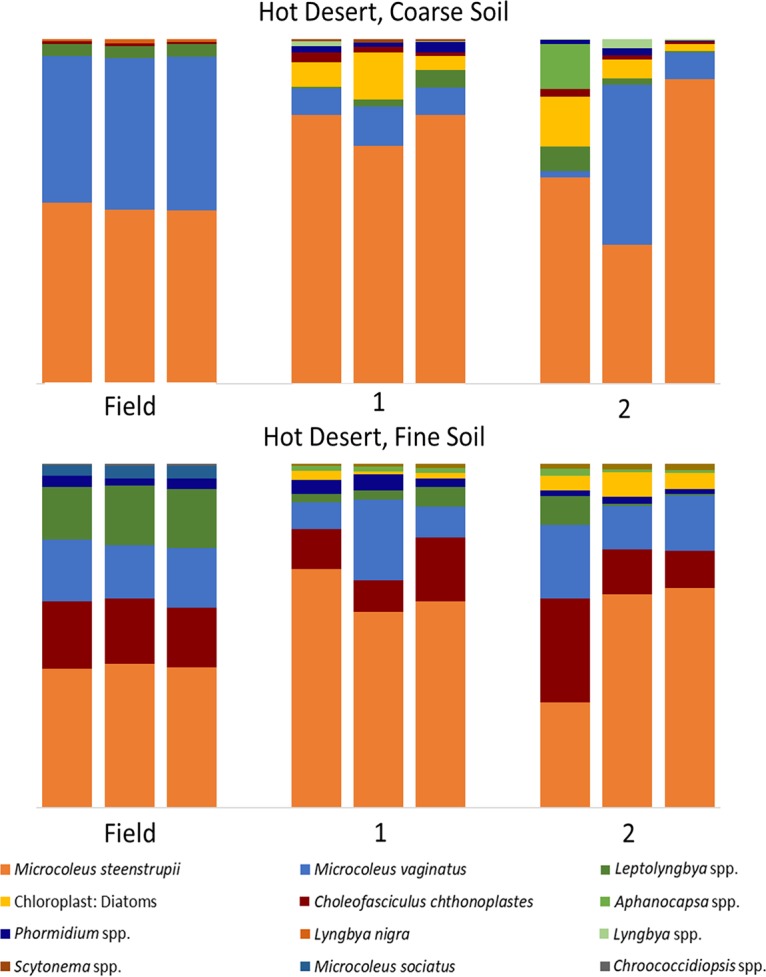
Cyanobacterial community composition in hot desert biocrusts, based on 16S rRNA amplicon sequencing, in biocrusts collected from the field (field), from the phase A incubation (1), and those resulting from a second recurrent production (2).

**FIG 3:**
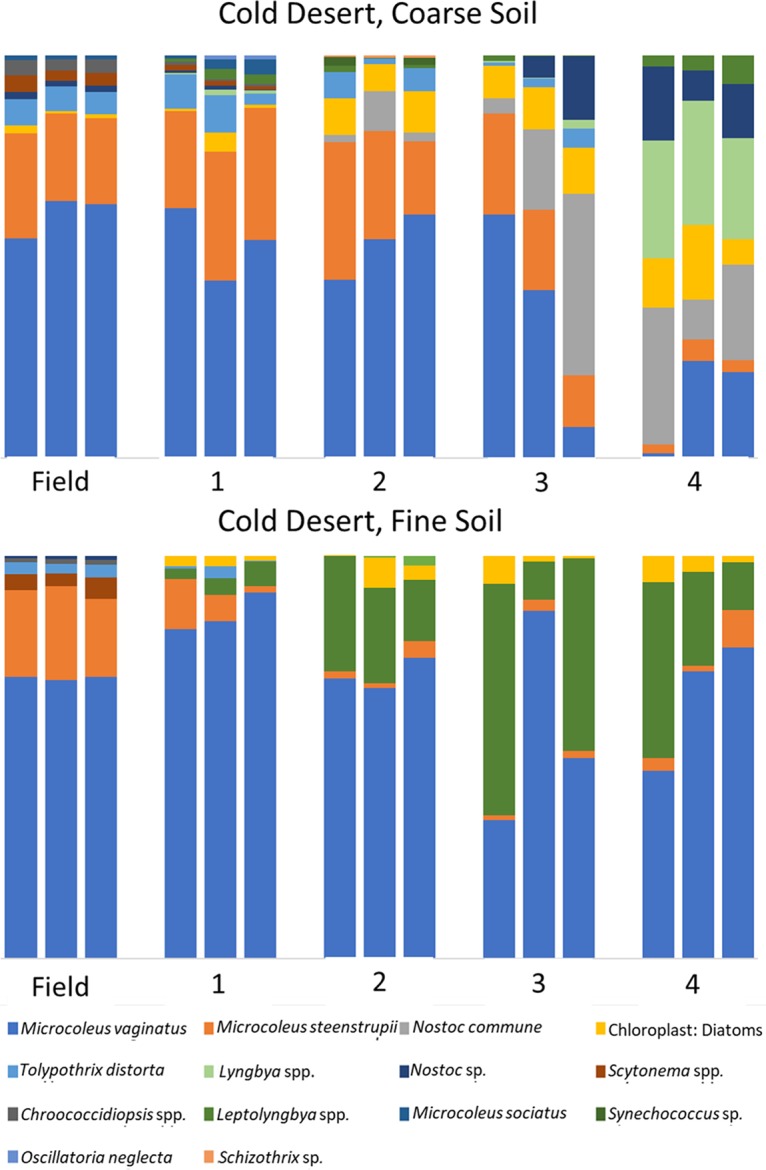
Cyanobacterial community composition in cold desert biocrusts, based on 16S rRNA amplicon sequencing, in biocrusts collected from the field (field), from the phase A incubation (1), and those resulting from recurrent production according to round (2, 3, and 4).

**FIG 4:**
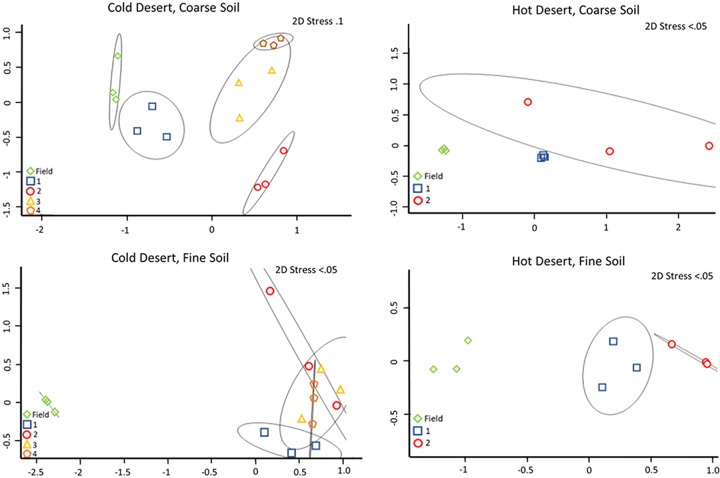
2D MDS of cyanobacterial community composition based on 16S rRNA amplicon sequencing, in biocrusts collected from the field (field), from the phase A incubation (1), those resulting from recurrent production according to round (2), and for cold desert biocrusts (3 and 4).

An analysis of the phototrophic community structure of nursery-produced biocrusts, run on the basis of taxa, revealed minimal shifts in the dominant species for the first growth round (Bray-Curtis, pairwise permutational multivariate analysis of variance [PERMANOVA], *P* = 0.1) in at least three of the soils (cold coarse, hot fine, and cold fine). This is consistent with the results obtained in similar experiments by Velasco Ayuso et al. (32). In the case of the hot coarse crust with complex growth dynamics, there were significant relative decreases (from 45% to 9%) already at the end of the first growth period for Microcoleus vaginatus (analysis of variance [ANOVA], *P* < 0.001), while Microcoleus steenstrupii increased in relative abundance from 51% to 75% (ANOVA, *P* = 0.004). Given that the average chlorophyll *a* level decreased from 46.1 mg m^−2^ to 3.0 mg m^−2^, this implies absolute losses for both populations. New taxa, including diatoms, undetectable in the original samples, also became important components (Fig. 2). In the cold desert incubations, where recurrent incubations yielded good growth, there were progressively more marked shifts in species compositions at each round (Fig. 3 and 4). The community compositions in the final round of growth of all biocrust types differed from their respective field community compositions based on Bray-Curtis similarity indices of relative abundance (Bray-Curtis, mains PERMANOVA, *P* = 0.003, cold fine; *P* = 0.004, cold coarse; *P* = 0.02, hot coarse; *P* = 0.03, hot fine) (Fig. 4). For example, *Lyngbya* spp. and Nostoc commune became more prevalent in cold desert coarse soils in the final growth round (ANOVA, *P* < 0.001, *P* = 0.005, respectively), contributing 18.2% and 14.4% of the dissimilarity, respectively, whereas *Leptolyngbya* spp. became more prevalent in the fine soil of cold desert sites (ANOVA, *P* = 0.04), contributing 39.3% of the dissimilarity as assessed by similarity percentage (SIMPER) analysis. Diatoms increased overall in both cold desert soils and hot desert fine soil (ANOVA, *P* = 0.03, coarse soil; *P* = 0.04, fine soil; *P* < 0.001, hot desert fine soil). These changes were also observed by microscopic inspection. For coarse hot desert biocrust, the interpretation was more difficult, in that in the absence of net phototroph population growth, shifts must have accrued through differential survival rather than by differential growth (Fig. 2). A detailed assessment of shifts based on phylotype (operational taxonomic unit [OTU]) for organisms identified at the genus level, such as *Leptolyngbya* spp., *Lyngbya* spp., *Scytonema* spp., *Chroococcidiopsis* spp., and *Nostoc* spp., confirmed patterns observed at the taxon level, with significant shifts in community composition (mains PERMANOVA, *P* < 0.001, cold fine and coarse; *P* = 0.003, hot fine; *P* = 0.004, hot coarse) (see Fig. S2, S3, and S4 in the supplemental material).

Two key species, *Microcoleus vaginatus* and *Microcoleus steenstrupii*, are of special interest as they are the pioneer (7) and dominant biocrust organisms (34). Within hot desert biocrusts, *M. steenstrupii* was typically a dominant community member and continued to dominate in both soil types throughout all growth rounds. Community shifts primarily introduced increased diversity in secondary community members but also losses of *Scytonema* spp. and Microcoleus sociatus (Fig. 2). In cold deserts, where *M. vaginatus* is typically dominant, it remained dominant in nursery-produced biocrusts for at least 3 rounds of growth in coarse soil and for the whole treatment in fine soil (Fig. 3).

### Pathogenic agents.

**FIG 5:**
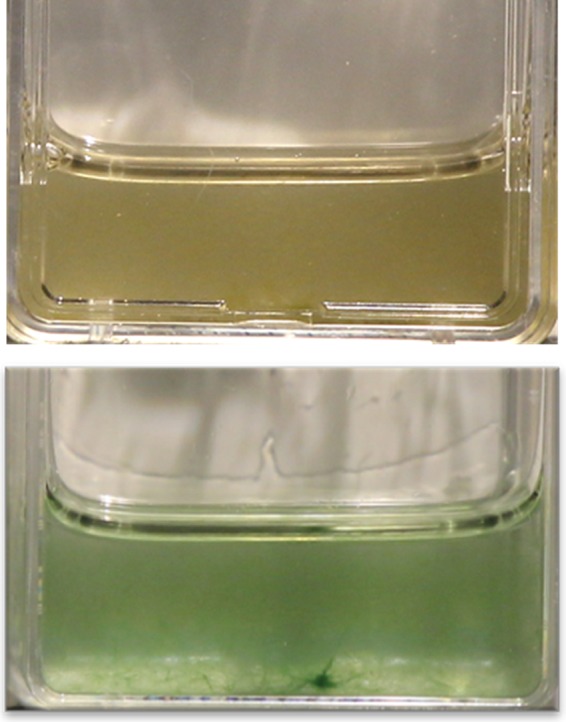
Bioassay determination of virulence toward *M. vaginatus* PCC9802. Liquid cultures were inoculated with 0.2 g of biocrust soils, and the potential to kill *M. vaginatus* was determined visually after 5 and 12 days of incubation. Top photograph is a positive for virulence, bottom is a negative for virulence.

Given the unexpected growth dynamics, we tested for the presence of pathogenic agents of cyanobacteria in our biocrusts. Bioassays of soil pathogenicity to *Microcoleus vaginatus* PCC9802 (Fig. 5) in phase A containers resulted in mortality of 70% to 80% in assays from hot soils, 60% of cold coarse soil samples, but only 10% in those from cold fine soil (Table 1). No virulence was recorded in any of the assays inoculated with autoclaved soils, and uninoculated controls remained healthy in all cases. Additionally, size filtration indicated the pathogen ranged in body size from 0.45 μm to 0.8 μm, excluding most viral and eukaryotic predators or pathogens (35). It is likely bacterial in nature.

**TABLE 1:** Virulence of biocrust according to *M. vaginatus* killing assay

Location	Soil type	Treatment	No. of replicates	No. of samples^*a*^	
				Strong virulence	Weak virulence
Hot (NM)	Coarse	Autoclaved	3	0	0
		Live	10	8	0
	Fine	Autoclaved	3	0	0
		Live	10	7	0
Cold (UT)	Coarse	Autoclaved	3	0	0
		Live	10	4	2
	Fine	Autoclaved	3	0	0
		Live	10	1	0

a Strong virulence denoted by death at 5 days of incubation, weak virulence by death at 12 days.

## DISCUSSION

With respect to our first goal, a strict reading of our results obtained here would be that growth dynamics are heterogenous but appear to be dependent on biocrust inoculum origin, including climatic aspects, whereby biocrusts from cold deserts took 3 to 7 weeks to reach maximum levels whereas biocrusts from hot desert took roughly twice as long (12 weeks). Our data indicate that cold biocrusts will likely develop photosynthetic biomass at a higher rate than warm biocrusts, possibly due to longer active periods enabled by more frequent wetting: warm biocrusts were only watered every 3 days whereas cold desert biocrusts were watered every 2 days. Also, significantly warmer conditions for hot desert biocrust also generally result in faster soil water evaporation. Since soil biocrusts are only active when wet (36, 37), these two factors may have caused a much longer (approaching double) cumulative growth period for the hot desert biocrusts. This could explain the differences in biocrust growth in the two desert soil types. Of course, simulating the temperature and wetting frequency of the local climatic regime is considered crucial in maintaining the original community composition, and maintaining a stable community composition should be a priority, as there is evidence of biogeographic patterns in the distribution of biocrust microbes (34, 38, 39) and inoculation with foreign microbes may introduce invasive species or low-quality inoculum. We did not observe a strong effect of soil texture on growth dynamics, although previous field studies have shown a positive correlation between finer textured soil and an increase in biocrust cover (40). Based on these results, shortening the times for the original protocols, duplicated here (which were around 8 weeks in duration [32]), to 3 to 4 weeks seems like a feasible optimization for production of biocrusts from cold deserts, particularly since the community structure remained rather stable, conserving significant populations of the major biocrust components. In the case of biocrusts from warm deserts, the original 8-week growth duration (32) seems already to have been close to optimal.

However, the previous data interpretation would obviate the unexpectedly high level of variability in growth among replicates. It far exceeded that of analytical error and suggests that the variability was in the organisms themselves. It is clear that for at least some replicates (usually seen as outliers in Fig. 1), very fast biocrust growth is possible, but these growth rates were not realized in all replicates. Apparently, stochastically distributed and important loss factors to the phototrophic populations were at play; hence, the dynamics failed to conform to simple models of a linear (or exponential) growth dynamics followed by stasis at carrying capacity. Previous nursery production of restoration inoculum had similar results, with high levels of growth in some containers and little to none in others; however, containers with high levels of growth were used for restoration inoculum and containers with poor growth were excluded (Corey Nelson, personal communication). This approach was likely an unintentional selection against any native loss factors. This level of complexity, and the nature of those loss factors, must be studied and understood, not only to better understand biocrust ecology in its basic sense, but also for an effective application in the production of inoculum for restoration: being able to suppress those loss factors would lead to maximal growth rates in every growth container. The nature of those loss factors is as yet unknown, and the literature does not offer much solace. With respect to grazing by microinvertebrates, several publications have documented their presence in soil biocrusts (41–46) even though grazing pressure has not been measured as a significant ecological factor, and there is no evidence that those microinvertebrates may be stochastically distributed. Alternatively, disease can be a significant loss factor in the population dynamics of phototrophs. Examples can be found in the literature on the severe impact of cyanophages on the population dynamics of planktonic blooms of cyanobacteria (47–50), and soils are known to harbor significant populations of bacteriophages (51, 52). However, also in the case of infectious agents, we would need to find one that is rare enough as to appear stochastically at the centimeter scale. The unidentified plaque-forming agent described by Sorochkina et al. (53) in similar biocrusts might be a potential contender. A retrospective examination of this possibility via bioassays with relevant cyanobacterial cultures clearly demonstrated the potential for virulence of the nursery biocrust soils. Hot desert biocrusts, which were particularly virulent, presented the most variable dynamics and the weakest yield. The presence of this biological agent(s) likely had a profound impact on biocrust growth, and the homogenization of inoculum that was carried out in phase A and again in phase B probably contributed to its spread. An effort to establish the identity via 16S rRNA gene amplicon sequencing was not successful due to increasing numbers of soil heterotrophic bacteria following cyanobacterial death; studies of the isolation, etiology, and relevance in natural communities of this cyanobacterial pathogen are under way. Until that research is completed, it is our recommendation that a process of bioassay testing of inoculation be carried out prior to nursery incubations, selecting at each step only pathogen-free containers and avoiding cross-container inoculum homogenization.

With respect to our second goal to establish if the recurrent use of inoculum was feasible in nursery biocrust production, we can safely say that in our treatments, this was not an advisable strategy for cold desert biocrusts. While it was possible in some cases to reuse inoculum in a second, or even third, incubation without major community shifts, there was evidence for a clear cumulative divergence (Fig. 3 and 4). Based on our treatments, if recycled inoculum is used, we would suggest it be closely monitored. Beyond the issue with community structure, the cumulative lack of fitness for growth of the recycled inoculum in warm deserts was rather unexpected. If our ideas with respect to the importance of randomly distributed deleterious agents as the cause of sluggish and inconsistent growth hold, then the homogenization of the inoculum from prior recurrent inoculation may have in fact ensured the presence of those agents in all phase B containers and the eventual cessation of growth in all replicates. This risk was clearly unanticipated and provides a warning for mixed-community approaches, adding to the advantages of biocrust inoculum production through mass cultivation of isolated biocrust organisms (33). Caution must also be exercised when choosing the source inoculum from the field. Minimally, inoculum should appear healthy. At the very least, careful monitoring of the growth trends of nursery biocrusts will allow any “diseased” biocrusts to be removed from production.

## MATERIALS AND METHODS

### Field locations and sampling.

We collected four types of remnant biocrust that differed in substrate soil type and climate/region of origin, as well as bulk soil from each of the locations. Hot desert coarse soils (loamy sand) were from the Chihuahuan Desert (Fort Bliss military base, Texas; lat 32.431069°, long −105.984151°) and hot desert fine soils (clay loam) from New Mexico (Jornada Basin long-term ecological research site; lat 32.545580°, long −106.723240°). Cold desert coarse soil (sandy clay loam, lat 41.104198°, long −113.008204°) and fine soil (clay loam, lat 41.104211°, −113.008204°) were collected from the Great Basin Desert, at Hill Air Force Base, Utah, test and training range. Hot desert crusts were at a level of development (LOD) (54) of 1, a light cyanobacterial crust, and cold desert crusts at an LOD of 6, with pedicels ranging from 0.5 mm to 7.5 mm. Sampling and storage protocols were according to those described by Velasco Ayuso et al. (32).

### Greenhouse incubations.

Greenhouse incubations were carried out in discrete, 12-cm by 12-cm by 5-cm Tupperware-type plastic containers containing bulk native soil (250 ml) that was used as the filler in the containers upon which the biocrust was inoculated. Temperature and watering regimes roughly mimicked those of the sites of origin: hot desert conditions were simulated at an Arizona State University greenhouse in Tempe, Arizona, from October 2016 through April 2017 with an average outdoor temperature of 23.1°C (ranging from 3.9°C to 37.2°C) (U.S. Department of Commerce, National Weather Service), and cold desert conditions were simulated at a Northern Arizona University greenhouse in Flagstaff, Arizona, from November 2016 through May 2017 with an average indoor temperature of 16.6°C (ranging from 12.5°C to 30.2°C) (U.S. Department of Commerce, National Weather Service). Each container then served as a sacrificial independent sample for our time course experiments. A wicking watering system (55) was used to avoid flooding while wetting the biocrusts. Deionized water was delivered to 80% soil holding capacity every 2 and 3 days for hot and cold desert locations, respectively, allowing the soil to dry naturally. To optimize growth, test containers received initial nutrient supplements, delivered as either 4.7 ml each of an N+P solution (357 mM NH_4_NO_3_, 80.5 mM K_2_HPO_4_, 80.6 mM KH_2_PO_4_ in double deionized water) or a P-only solution (80.6 mM KH_2_PO_4_ in double deionized water) (see Fig. S1 in the supplemental material for details). Whether to supplement a soil with both N and P or just P was according to prior determinations (32). For inoculation, natural biocrusts from the corresponding location were gently crushed, homogenized, and spread over the surface of the bulk native soil so as to inoculate at roughly 5% of the population density (based on areal chlorophyll *a*; see below) in the original field biocrusts. Cloth that reduced incoming solar radiation by 60% was placed on top of each of the containers and positioned approximately 2 cm above the soil surface (32).

### Experimental design.

We set up 4 treatments, one for each of our four field biocrusts using its bulk native soil, each having two phases. First, we followed biomass development weekly (phase A), to later probe the potential to use the biomass obtained in phase A to serve as inoculum for successive rounds of growth (phase B). Each container was used as an independent harvested sample, and three containers were randomly sampled per time point. Additional containers containing bulk native soil were incubated, watered, and supplemented with nutrients but left uninoculated, as controls for growth based on airborne cyanobacterial propagules rather than from the inoculum (53).

Biocrusts obtained during phase A were pooled and used as inoculum for phase B, in which the biomass resulting from a round of growth (4 or 8 weeks of growth in the nursery) was used to seed the next round. To accelerate phase B, inoculation levels for cold desert sites (rounds of growth 3 and 4) and hot desert sites (all rounds of growth) were increased to the equivalent areal chlorophyll *a* cover of 15% of that existing in the biocrust used as inoculum. A flow chart is provided (Fig. S1) for tracking treatment details, including inoculation levels, growth rounds, sampling, and time points.

### Biomass determination.

Chlorophyll *a* areal concentration was used as a proxy for photosynthetic biomass. Biocrust cores (0.5 cm deep, 0.5 cm diameter) were collected and kept at 4°C in dark under dry conditions until analysis. Two cores were collected per container, yielding a total of 6 replicates per time point. Chlorophyll *a* was extracted in the dark at 4°C for 24 h following the Giraldo-Silva method described by Sorochkina et al. (53), after sample grinding by mortar and pestle in 90% acetone. The centrifuge-clarified samples (8,437 × *g* at 15°C for 10 min) were analyzed spectrophotometrically in a Shimadzu UV1601 spectrophotometer, according to the protocol of Garcia-Pichel and Castenholz (56), which corrects for scytonemin and carotenoid interference.

### Bioassay for presence of cyanobacterial pathogens.

Axenic liquid cultures of *Microcoleus vaginatus* (PCC9802) (1), grown in Jaworski’s medium (JM) (57), were inoculated with 0.2 g of biocrust from 10 randomly selected phase A containers for each crust type. Two sets of liquid *M. vaginatus* (PCC9802) cultures served as controls: one was inoculated with 0.2 g of autoclaved soil from each location, and a second set was left uninoculated. Health was visually monitored for 2 weeks, with healthy filaments appearing green and diseased or dead cells appearing brown. Biocrust inoculated cultures that resulted in the death of *M. vaginatus* were syringe filtered (0.2 μm, 0.45 μm, 0.8 μm, 1 μm, and 3 μm) and inoculated in healthy *M. vaginatus* cultures. *M. vaginatus* health was tracked to approximate the pathogen’s body size.

### Cyanobacterial microbial community structure.

A first screening for key community members was performed from additional discrete samples by bright-field microscopy using a compound microscope (Nikon Labophot-2). Additionally, biocrust cores (0.5 cm deep, 0.3 cm diameter) were taken for assessment via 16S rRNA gene amplicon. Field biocrusts used to seed phase A growth were sampled in triplicates, and nursery grown biocrusts were sampled in replicates of 9 at the end of each growth round for each of the 4 biocrust types. All cores were stored at −80°C until the bacterial community composition was determined via high-throughput Illumina sequencing of PCR-amplified 16S rRNA gene amplicons. The cores for each growth round were randomly pooled and homogenized into three composite samples, so that three independent sequencing reactions per growth round and biocrust type were run. DNA was extracted from the three composite samples via the Qiagen DNeasy PowerSoil kit, reference number 12888-100, according to the manufacturer’s instructions. The V4 region of the 16S rRNA gene was amplified using the barcoded primer set 515F and 806R (58). Triplicate PCRs included the following: denaturation at 94°C for 3 min, 35 cycles of denaturation at 64°C for 45 s, annealing at 50°C for 50 s, and extension at 72°C for 90 s, and a final extension at 72°C for 10 min. PCR amplifications for each composite sample were pooled, and DNA yield was quantified with a Quant-iT PicoGreen dsDNA assay kit (Life Technologies, NY, USA). Two hundred forty nanograms of DNA per sample was used for library preparation after purification via a QIAquick PCR purification kit (Qiagen, Valencia, CA, USA). The DNA concentration of the PCR pooled library was quantified by the Illumina library quantification kit ABI Prism (Kapa Biosystems, Wilmington, MA, USA). The PCR pooled library was diluted to a final concentration of 4 nM and denatured before being mixed with 30% (vol/vol) of 4 pM denatured Phi X viral DNA. Finally, the PCR pooled library and Phi X mixture was loaded in the MiSeq Illumina sequencer cartridge, and the run was performed using chemistry version 2 (2 × 150 paired end) according to the recommendations of the manufacturer (Illumina, San Diego, CA, USA). Sequencing was performed in the Microbiome Analysis Laboratory at Arizona State University (Tempe, AZ, USA), yielding raw FASTQ sequence files.

### Bioinformatic analysis.

The raw FASTQ file was demultiplexed within the MiSeq Illumina workflow under default parameters. Paired sequences were demultiplexed and analyzed via Qiime2.10 (59), using the DADA2 plugin (60) to create a feature table with representative sequences (features) and their frequency of occurrence. To remove highly variable positions, sequences were aligned with the MAFFT program (61). FastTree (62) was used to generate a tree. Taxonomy was assigned with the Naive Bayes classifier trained on the Greengenes 13.8 release, where sequences were trimmed to include 250 bases from the V4 region, bound by 515F/806R primers (58). We chose to focus on cyanobacteria, because they are the pioneers of biocrust formation (7, 8) and thus an optimal target for restoration. As such, we selected them from the master feature table using the filter_taxa_from_otu_table.py function in Qiime1 (58). Given the poor taxonomic resolution obtained with Greengenes (63), cyanobacterial sequences that attained at least 0.005% of the total number of cyanobacterial features were then phylogenetically assigned using our own curated cyanobacterial database/tree version-0.22 (https://github.com/FGPLab/cydrasil/tree/0.22a) via RAxML (64) and displayed using ITOL (65).

### Statistical analyses.

In phase A, we define peak biomass as the largest average chlorophyll *a* concentration obtained at any one point. Statistically significant growth is defined as a significant difference between chlorophyll *a* concentrations at peak biomass and those at inoculation. Welch’s *t* tests were used for all controls and biocrust types except for the cold desert fine soil treatment, where the nonparametric Kolmogorov-Smirnov test was used because of unequal variances. For microbial community analyses, significance in composition shifts was tested with mains PERMANOVA calculated on Bray-Curtis similarity matrices of relative abundances derived from sequencing with 9,999 permutations and visualized using two-dimensional (2D) multidimensional scaling (MDS) plots. Ellipses indicate 90% confidence intervals. All calculations were performed using R (66) except for shifts in specific cyanobacterial community members. Shifts in specific cyanobacterial community members (as taxa, not OTUs) were analyzed using one-way analysis of similarity (ANOSIM) with 9,999 permutations combined with SIMPER analyses using relative abundance of taxa (not OTUs). Taxa identified, via SIMPER, as drivers of dissimilarity were assessed for significant shifts using ANOVAs, within the PRIMER software, v6 (67).

### Data availability.

Raw sequence data have been submitted to NCBI and are publicly available under the BioProject number PRJNA515304.

## Supplementary Material

Supplemental file 1
