# Supplementary material for: Optimizing the Production of Nursery-Based Biological Soil Crusts for Restoration of Arid Land Soils
Source: Appl Environ Microbiol. 2019 Jul 18;85(15):e00735-19. doi: 10.1128/AEM.00735-19 (PMC6643228; doi:10.1128/AEM.00735-19)
Supplement: Supplemental file 1 [file AEM.00735-19-s0001.pdf]

Supplemental Material

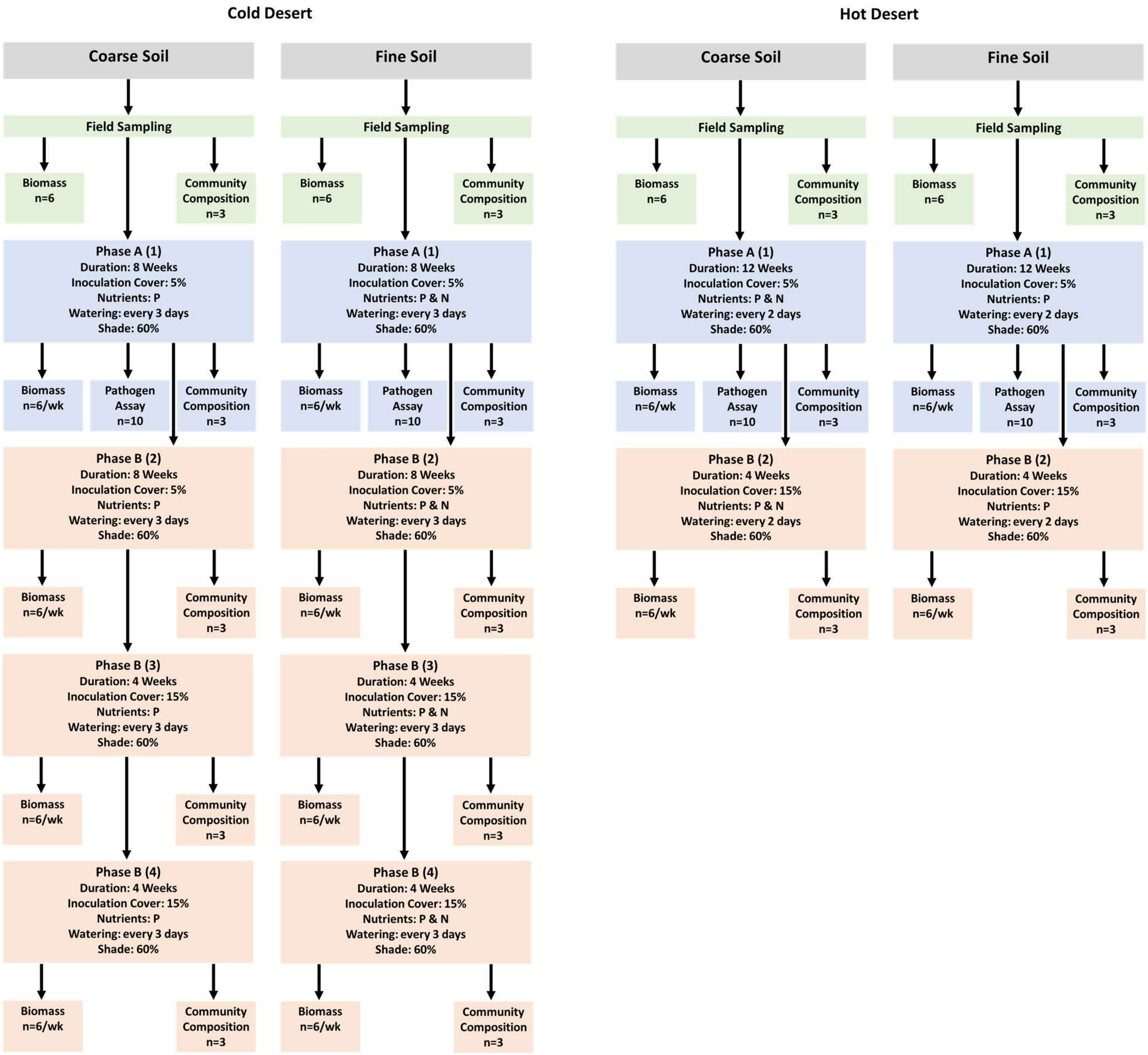

Supplementary Fig 1. Experimental flow chart including inoculum levels, growth rounds, sampling and time points. Field biocrusts are shown in green. Phase A is shown in blue and Phase B is shown in orange.

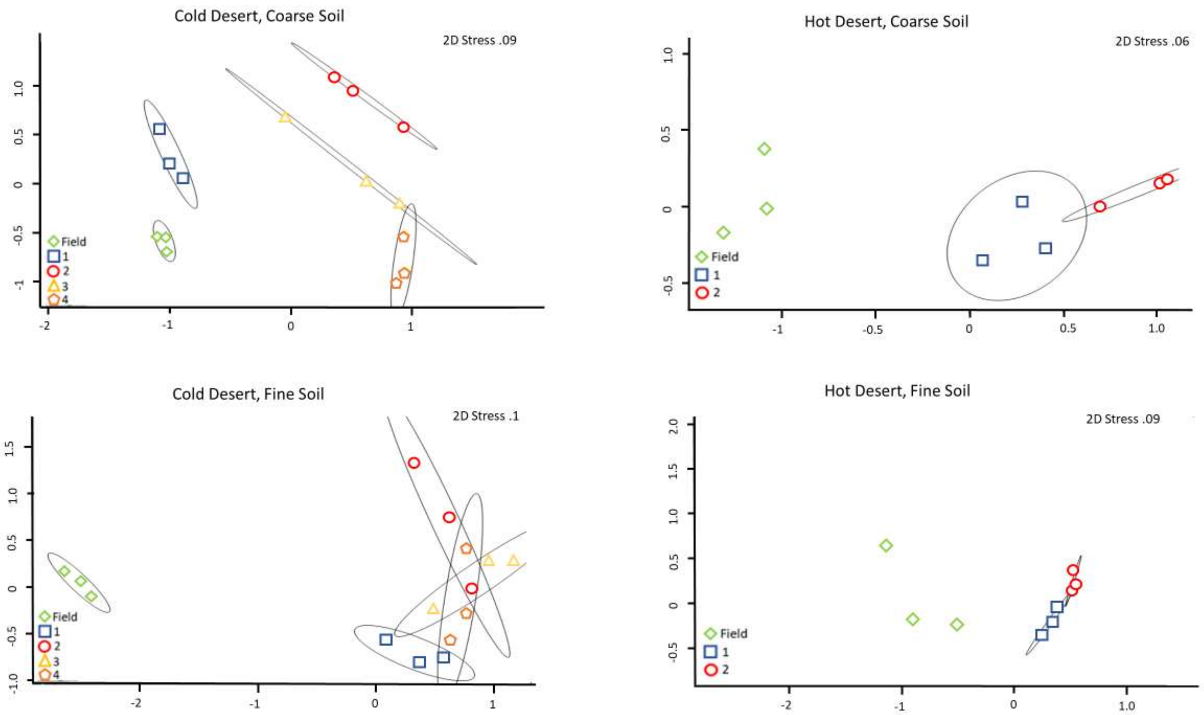

Supplementary Fig 2. 2-D MDS of cyanobacterial community composition, similar to Fig. 4, in which the taxonomic resolution for cyanobacteria has been increased to the maximum possible. Legend: biocrusts collected from the field (Field), from the end of Phase A incubation (1), and those resulting from recurrent production in Phase B, according to round (2, 3, and 4).

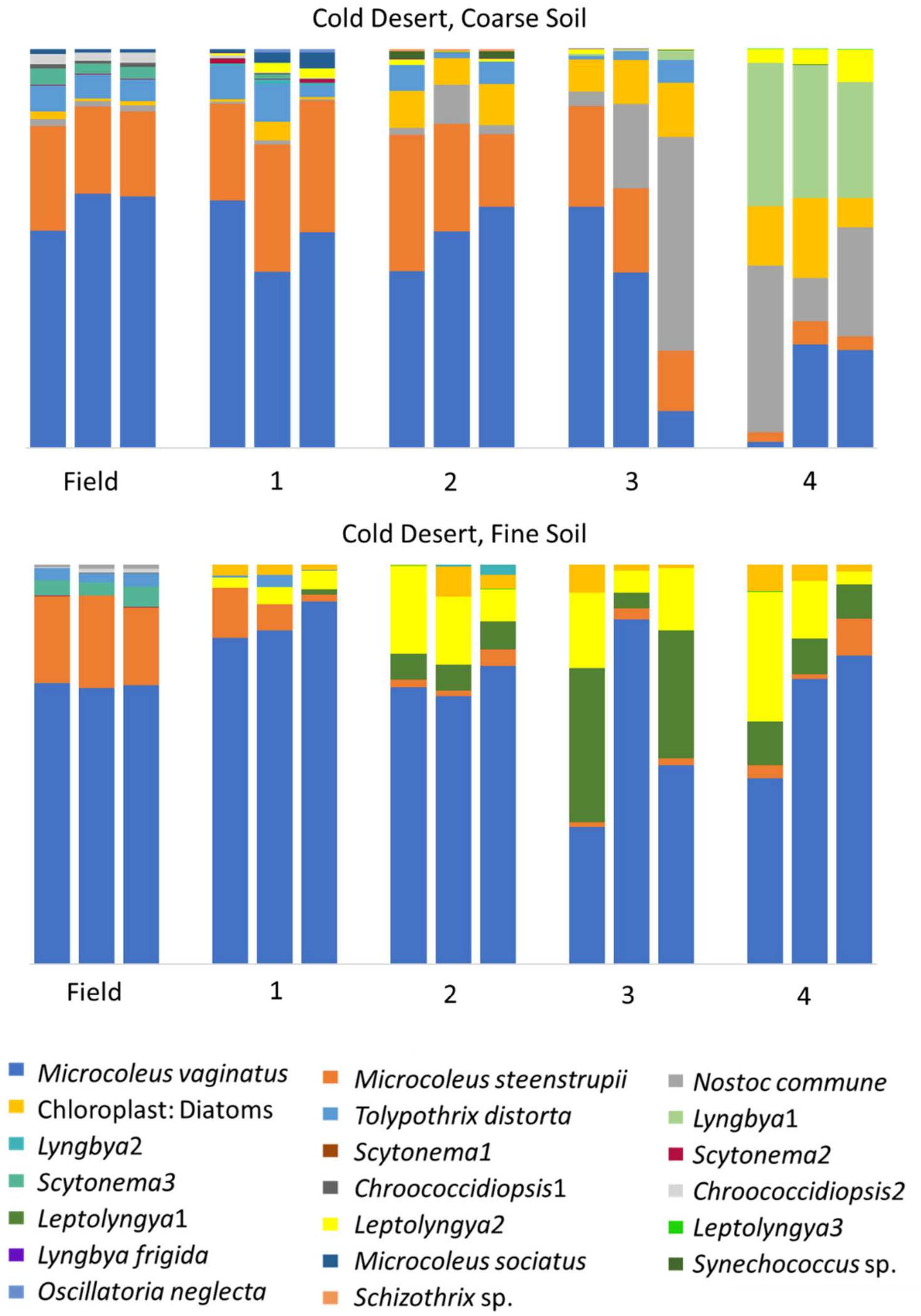

Supplementary Fig 3. Cyanobacterial community composition at the species, or phylotype level where species identification was not possible, in cold desert biocrusts, based on 16S rRNA amplicon sequencing. Communities shown include biocrusts collected from the field (Field), from the end of Phase A (1), and those resulting from recurrent production in Phase B according to round (2, 3 and 4).

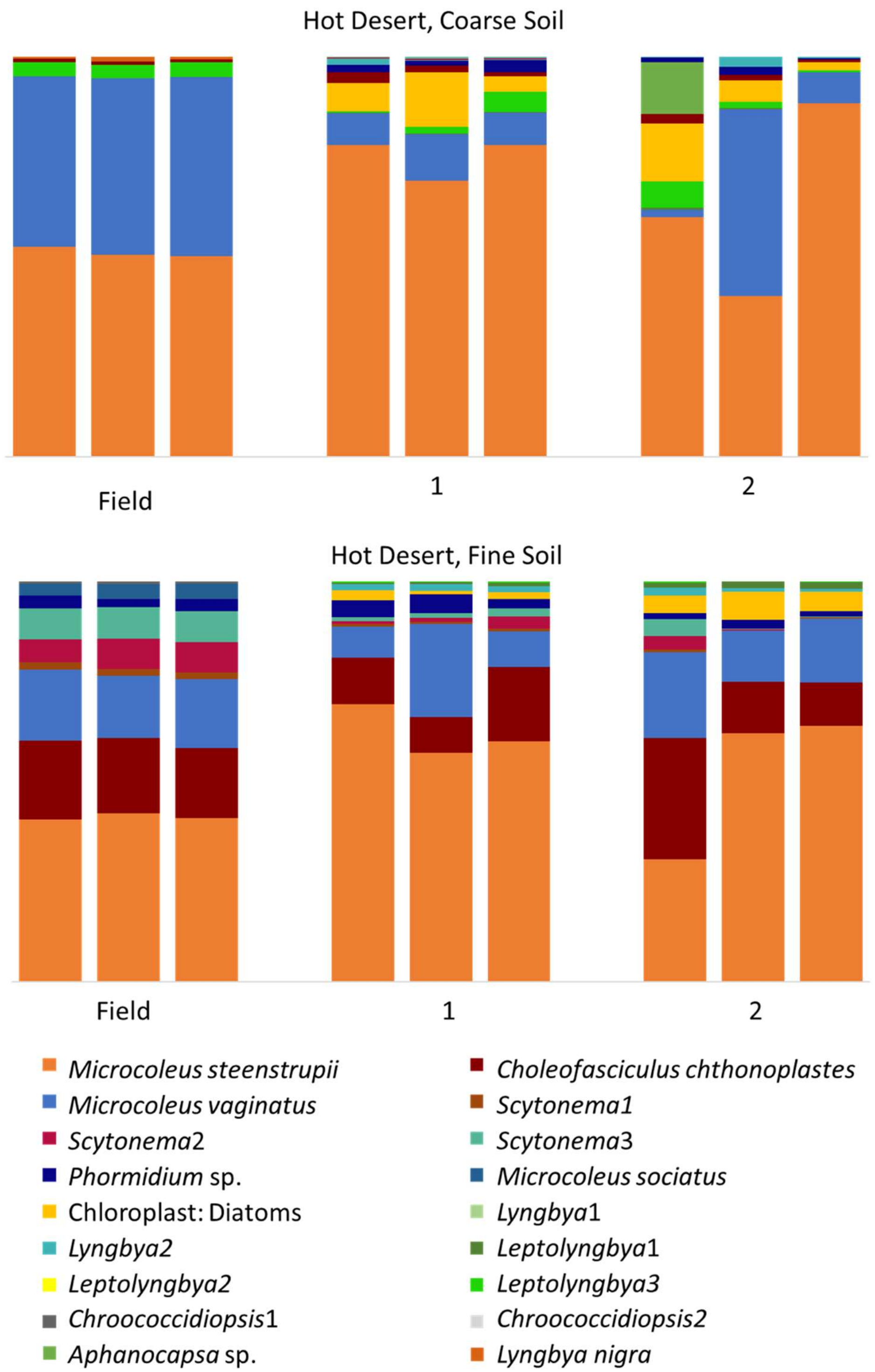

Supplementary Fig 4. Cyanobacterial community composition at the species, or phylotype level where species identification was not possible, in hot desert biocrusts, based on 16S rRNA amplicon sequencing. Communities shown include biocrusts collected from the field (Field),

from the end of Phase A (1), and those resulting from recurrent production in Phase B according to round (2).
